# A comparative study: Influence of various drying methods on essential oil components and biological properties of *Stachys lavandulifolia*

**DOI:** 10.1002/fsn3.2218

**Published:** 2021-03-08

**Authors:** Saeid Hazrati, Kazem Lotfi, Mostafa Govahi, Mohammad‐Taghi Ebadi

**Affiliations:** ^1^ Department of Agronomy Faculty of Agriculture Azarbaijan Shahid Madani University Tabriz Iran; ^2^ Department of Nano Biotechnology Faculty of Biotechnology Amol University of Special Modern Technologies Amol Iran; ^3^ Department of Horticultural Science Faculty of Agriculture Tarbiat Modares University Tehran Iran

**Keywords:** active constituents, antioxidant activity, aromatic plants, drying

## Abstract

The genus *Stachys* is a member of the Lamiaceae family. These are important medicinal plants which grow all over the world and are known for their flavoring and therapeutic effects and *Stachys lavandulifolia* is an endemic species of Iran. To acquire high‐quality essential oil (EO), drying technique was implemented which is an essential part of this process. The present study designed to evaluate the influences of different drying techniques (fresh sample, shade, sunlight, freeze‐drying, microwave, and oven‐drying (40, 60, and 80°C) on EO yield and composition of *S. lavandulifolia*. The results indicated that the maximum EO yield was obtained by the shade‐drying method. The main compounds found in the fresh samples were spathulenol, myrcene, β‐pinene, δ‐cadinene, and α‐muurolol, while spathulenol, cyrene, δ‐cadinene, p‐cymene, decane, α‐terpinene, β‐pinene, and intermedeol were found to be the dominant compounds in the dry samples. Drying techniques were found to have a significant impact on the values of the main compositions, for example, monoterpene hydrocarbons such as α‐pinene, β‐pinene, myrcene, and β‐phellandrene were significantly reduced by microwave drying, oven‐drying (40, 60, and 80°C), and sunlight‐drying methods. Drying techniques increased the antioxidant activity of *S. lavandulifolia* EOs especially those acquired by freeze‐drying with the half‐maximal inhibitory concentration (IC_50_) values 101.8 ± 0.8 mg/ml in DPPH assay and 315.2 ± 2.1 mg/ml in decreasing power assay. As a result, shade‐, sun‐, and oven‐drying (40°C) were found to be the most important techniques for attaining maximum yields of EO.

## Graphical abstract

The present study designed to evaluate the influences of different drying techniques (fresh sample, shade‐drying, sunlight‐drying, freeze‐drying, and oven‐drying (40, 60, and 80°C), and microwave‐drying) on EO yield and composition of *Stachys lavandulifolia*.

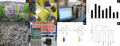

## 1 INTRODUCTION

Iran is one of the most important centers of plant diversity in the world. Nearly 22% of the 8,000 plant species found among the flora of Iran are native (endemic). *Stachys lavandulifolia,* which belongs to *Lamiaceae*, is an endemic and perennial flowering plant, which grows as woody shrubs on mountainsides of Iran. Its flowers are arranged as spikes, similar in appearance to those of cotton, and range from blue to purple in color (Jalilian et al., 2013). In traditional medicine, because of their effective traits in curing illnesses, the genus *Stachys* plants has been used (Bahadori et al., 2020), particularly in the treatment of ulcers, cough, and sclerosis of the spleen, inflammation, and genital tumors (Tundis et al., 2014; Zargari, 1995). In Iran, *S. lavandulifolia* is traditionally used to treat gastrointestinal disorders in the form of an herbal tea, while its extract has been applied as an anxiolytic and mild sedative which has been compared with diazepam (Amin, 1991).

Drying is the most common method of storing medicinal and aromatic plants and protecting their biochemical compounds (Rahimi & Farrokhi, 2019). Medicinal plants in the postharvest stage are very sensitive to fungal damage, due to their high moisture content. Therefore, in choosing the most appropriate method, the drying moisture should be reduced by 10%–12% (Azizi et al., 2010; Brovelli et al., 2003). In recent decades, many studies have been conducted on herb‐drying, and several new methods have been introduced to the field. Studies over the past 20 years have focused on drying methodologies and techniques have been established to increase quality as well as to enhance the efficiency of the drying process (Mahmoudi et al., 2020; Thamkaew et al., 2020). The essential oils (EOs) of fresh plants are saved on leaves surfaces and in trichome which are specialized structures (Werker, 2000). Integrity of the oil glands in the dried products relies on the shelf life of EOs in dried leaves (Ebadi et al., 2015; Jangi et al., 2020). Thus, through conserving trichome integrity or reducing the damage to trichomes during drying and the aroma quality of dried herbs yield of EOs should be enhanced (Thamkaew et al., 2020).

*Stachys lavandulifolia* is a valuable endemic plant to Iran which has not been comprehensively studied in terms of the effects of various drying techniques on its volatile composition. The principal constituents of the *S. lavandulifolia* EO composition have been previously reported as α‐pinene, β‐pinene, germacrene‐D, and (Z)‐β‐ocimene (Bahadori et al., 2020). However, no studies have documented a suitable drying method for the preservation of volatile oil compounds in *S. lavandulifolia*. The antioxidant attributes of bio‐active molecules play a main role in preventing damage caused by free radicals. The working mechanism of some antioxidants will be changed by exposure to temperature variation, leading to reduction in antioxidant activity (Réblová, 2012).

Accordingly, the intentions of this study were: (a) to study the influence of seven drying techniques (shade‐, sunlight‐, freeze‐, oven (40, 60, and 80°C) and microwave‐drying) on the EO composition and yield in *S. lavandulifolia*, (b) to determine the best drying method in respect of maintaining the principal EO compositions of the *S. lavandulifolia* and, (c) to investigate the impact of various drying methods on the biological properties.

## 2 MATERIALS AND METHODS

### 2.1 Plant material

In June 2019, plant materials of *S. lavandulifolia* were collected from the Harris region situated in the East Azerbaijan province, Iran (38°56′N–45°37′E) circa 1,573 m above sea level. The specimens were recognized at the Research Institute of Forest and Rangelands by Flora Iranica.

### 2.2 Drying techniques and equipment

Considering that five types of drying methods (shade, sun, freeze, microwave, and oven‐drying) were used in this experiment, plant samples were divided into five groups to ensure the uniformity of plant materials in the treatments. In the case of shade‐drying, natural airflow, and ambient temperature were implemented (temperature = 26 ± 2°C). Where sun‐drying was used, the samples were dried under direct sunlight at temperature between 27°C and 37°C for 4 days in July in Tabriz, Iran. Freeze‐drying was performed in a laboratory freeze‐dryer for duration of 8 hr at −52°C. Microwave‐drying was carried out using a digital microwave oven at 600 W. Oven‐drying was carried out at two different temperatures (40, 60, and 80°C).

### 2.3 EO content

In order to extract the EO, dried aerial parts were used. To measure EO content, 50 g crushed samples were subjected to hydro‐distillation for 3 hr using an all‐glass Clevenger‐type apparatus. EOs were dried over sodium sulfate to calculate EO yield and stored at −20°C until analysis.

### 2.4 Gas chromatography

A gas chromatograph (Agilant 7890B) equipped with a flame ionization detector, and an HP‐5 capillary column (length 30 m, internal diameter 0.25 mm and 0.25 μm film thickness) were used for the analysis of EOs. Temperature plan contains 2 min at 60°C and enhancement to 250°C with a ramp of 5°C/min. Helium gas was used as the carrier at a flow rate of 1.1 ml/min in split ratio of 1:100.

### 2.5 Gas chromatography‐mass spectroscopy

The EO analysis was done using a GC equipped with MS detector (Thermo Quest‐Finnigan) and a 60 m × 0.25 mm, 0.25 µm fused silica column (DB‐5). The oven temperature was initially at 60°C, increased by 5°C/min for 38 min, and then maintained at 250°C for 10 min. Helium gas was used as a carrier gas with a flow rate of 1.1 ml/min. The splitting ratio was 1:100 and the injector and detector temperatures were adjusted at 250 and 280°C, respectively. The ionization voltage, scan time, and mass range were 70 eV, 0.4s, and 40–300 *m*/*z*, respectively. The EO constituents were determined using retention indices as well as Wiley and NIST 11.0 mass‐spectral libraries. The percentage of compounds was calculated by electronic integration of FID peak areas without the use of response factor correlation.

### 2.6 Evaluation of DPPH radical‐scavenging activity

DPPH radical scavenging of the EO was assessed pursuant to the technique introduced by Hazrati et al. (2019), with some minor changes. Briefly, 150 µl of the DPPH solution (0.1 mg/ml in methanol) was mixed with 150 µl of the EO at various amounts (250, 125, 62.5, 31.2, 15.6, and 7.8 µg/ml in methanol). The composition was incubated at 25°C for 30 min. Following this, the sample absorbance was measured at 517 nm. The following equation was used to measure the percentage of radical‐scavenging activity:

$$\mathrm{Adical}\;\mathrm{scavenging}\;\mathrm{activity}\;\left(\%\right)=\frac{A_{\mathrm{control}}‐A_{\mathrm{sample}}}{A_{\mathrm{control}}}\times 100$$

### 2.7 Reducing power determination

In this study, the potassium ferricyanide‐ferric chloride method was employed to evaluate the antioxidant activity of the EOs as stated by Tundis et al. (2014), with some modification. In addition, quercetin and methanol were used as a reference compound and negative control, respectively.

### 2.8 Statistical analysis

Statistical analysis was carried out with SAS 9.2 using one‐way ANOVA. Statistical significance of differences between means of yield and main components for EO was accepted at *p < *.05 by Duncan's multiple range test. In addition, Analytical data for Hierarchical Cluster Analysis was performed with SPSS version 25.0 software.

## 3 RESULTS AND DISCUSSION

### 3.1 Effect of drying techniques on essential oil content

**FIGURE 1:**
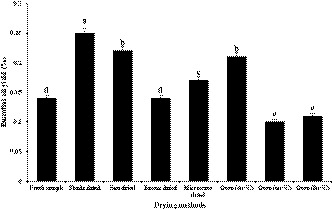
Effect of different drying methods on essential oil content of *Stachys lavandulifolia*. (Significant differences at *p* < .05 have been indicated by different letters)

The obtained results indicated that drying techniques had significant influence on EO content (Figure 1) and that the highest amount (0.25%) was acquired by shade‐drying. The lowest amounts of EO were acquired from oven‐drying at 60°C (0.10%) and 80°C (0.11%). However, there was no significant difference between the sun‐ and oven‐drying (40°C) methods. Thus, EO yield of shade‐dried plants was greater than that using other drying methods. With respect to different drying procedures, changes in the EO yield depend on the type of tissue temperature, time, and drying method employed (Dehghani Mashkani et al., 2018). Similar results were also reported by Ozdemir et al. (2017), who dried *Origanum vulgare* L. and *Origanum onites* L. in oven‐drying at 60°C, under shade and sunlight, and revealed that the highest EO yield was obtained by shade‐drying. Mokhtarikhah et al. (2020), demonstrated that EO yield of sun‐dried spearmint was higher than that of the fresh sample. Saeidi et al. (2016) indicated that EO yields of shade and oven‐dried (at 40°C) *Mentha longifolia* L. were greater than yields obtained by other drying methods. In research carried out on seeds (Rebey et al., 2020), the effect of various drying techniques on EO yield was studied and results indicated that EO amount in shade‐dried samples was more than that obtained by oven‐ and sun‐drying methods. Shade‐drying is, evidently, one of the most suitable methods for drying herbs. Because of the lower temperatures used in shade‐drying, the evaporation of fragrant compositions is lower, and amounts of EOs were found to be more than those samples subjected to oven‐ or sun‐drying (Mirhosseini et al., 2015).

### 3.2 Evaluation of drying techniques on EO compositions

As shown in Table 1, 21 chemical compositions were distinguished in the fresh and dried samples of *S. lavandulifolia*. The results showed that the major constituents in the fresh samples were spathulenol (21%), myrcene (13.5%), β‐pinene (9.8%), δ‐cadinene (9.8%), and α‐muurolol (9%). The results also clarified that regardless of drying methods, the main compositions remained spathulenol (0.6%–22.6%), cyrene (0.3%–17.5%), δ‐cadinene (0.1%–18.7%), p‐cymene (t–27.5%), decane (t–24.1%), α‐terpinene (t–18.4%), β‐pinene (0.3%–17.5%) and intermedeol (0.1%–12.5%).

**TABLE 1:** EO constituents (%) of *Stachys lavandulifolia* in different drying methods

No.	Compounds	RI	FS	SHD	SD	FD	MD	OD (40°C)	OD (60°C)	OD (80°C)
1	α‐Pinene	938	t	15.5	7.5	10.8	t	t	t	t
2	β‐Pinene	953	9.8	5	0.7	2.9	0.3	0.5	1.4	0.5
3	Myrcene	989	13.5	17.5	15	11.3	0.3	0.5	1.7	1.1
4	Decane	999	t	t	t	5.2	15	10.8	24.1	8.5
5	α‐Phellandrene	1,007	0.7	0.5	0.6	0.8	t	6.6	t	t
6	α‐Terpinene	1,022	0.7	0.8	0.2	0.1	12.6	t	18.4	6.4
7	p‐Cymene	1,030	t	t	t	t	16	7.5	27.5	27.3
8	β‐Phellandrene	1,033	5.3	2.9	0.7	3.8	0.3	0.7	0.4	0.3
9	α‐Copaene	1,391	4.5	1.2	3.8	13.7	1.2	5.6	t	1.9
10	E‐Caryophyllene	1,440	2.3	1.6	0.6	5.9	1.6	0.2	0.5	2.5
11	Sesquisabinene	1,464	0.2	1.9	3.5	0.6	0.4	0.3	0.4	1.2
12	Germacrene‐D	1,502	5.3	1.6	0.5	9.5	3.7	0.7	0.6	4.6
13	Bicyclogermacrene	1,517	2.3	2.5	0.2	0.1	2.7	0.1	0.2	1.8
14	δ‐Cadinene	1,538	9.8	10	7.5	18.7	0.9	10.6	5.1	0.1
15	Spathulenol	1,598	21.3	19.3	22.6	0.6	4	21.9	5.8	20.8
16	α‐Muurolol	1,662	9	1.9	0.6	7.5	1.9	0.7	0.8	7.3
17	α‐Cadinol	1,675	6	1.9	7.5	0.5	4.1	10.9	t	6.8
18	Intermedeol	1,684	0.6	1.9	12.5	0.3	1.6	0.4	0.1	0.1
19	Guaia‐3,10(14)‐dien‐11‐ol	1,693	3	2.5	5	0.6	4.2	7.5	t	t
20	epi‐α‐Bisabolol	1,699	0.1	0.6	5	0.2	5.2	6.6	0.7	0.3
21	(2Z,6E)‐Farnesol	1,735	0.3	0.6	0.9	0.4	8.9	2.1	t	t
Monoterpene hydrocarbons			30	42.2	24.7	29.7	29.5	15.8	49.4	35.6
Sesquiterpene hydrocarbons			24.4	18.8	16.1	48.5	10.5	17.5	6.8	12.1
Oxygenated sesquiterpenes			40.3	28.7	54.1	10.1	29.9	50.1	7.4	35.3
Other			—	—	—	5.2	15	10.8	24.1	8.5
Total			94.7	89.7	94.9	93.5	84.9	94.2	87.7	91.5

Note

RI: retention index, t: trace < 0.1%.

Abbreviation: FD, freeze‐dried; FS, fresh sample; MD, microwave‐dried; OD, oven‐dried; SD, sun‐dried; SHD, shade‐dried.

**FIGURE 2:**
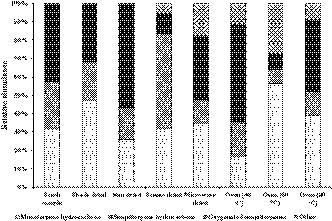
Comparison of main chemical groups (%) in *Stachys lavandulifolia* using different drying techniques

According to the results, the volatile compositions from *S. lavandulifolia* were divided into three chemical groups: monoterpene hydrocarbons, sesquiterpene hydrocarbons, and oxygenated sesquiterpenes (Figure 2). The results showed that sunlight‐, freeze‐drying, oven‐drying (at 40 and 80°C) and microwave‐drying significantly decreased amounts of hydrocarbon monoterpenes in comparison to shade‐drying. The most significant variations were observed in β‐pinene, α‐pinene, myrcene, and β‐phellandrene (Table 1). The highest α‐pinene value (15.5%) was obtained by shade‐drying, however, the α‐pinene amount reached its lowest level (t) when microwave and oven‐drying were employed. In addition, both sun‐ and freeze‐drying reduced α‐pinene amount (51.61 and 30.32%, respectively) when compared with shade‐drying. The most β‐pinene amount was acquired by shade‐ (5.0%) and freeze‐drying (2.9%) methods, however, the β‐pinene amount significantly reduced when microwave‐drying was used (0.3%). The highest myrcene content was acquired by shade‐ (17.5%) and sun‐drying (15.0%); however, myrcene content significantly reduced when microwave‐drying was used (0.3%) (Table 1). Both microwave‐ and oven‐drying (80°C) led to a significant decrease in β‐phellandrene values, reaching the least amount (0.3%) in comparison to shade‐drying (2.9%). However, the maximum β‐phellandrene amount (3.8%) was obtained when freeze‐drying was used. The total content of monoterpene hydrocarbons in the shade‐ and oven‐drying EOs (60 and 80°C) were higher than those in the fresh samples. When the drying temperature was enhanced in the microwave and oven, several monoterpene hydrocarbons were lost compared with methods at lower temperatures. Temperature‐sensitive compositions including a‐pinene, β‐pinene, myrcene, and β‐phellandrene, have a greater tendency to water fraction and evaporate with the water during the drying process (Hamrouni‐Sellami et al., 2012; Hazrati et al., 2018; Rahimmalek & Goli, 2013). In additional, the biological structure of EO‐producing glands is affected by higher temperatures and the epithelial cells are corrugated on each other (Diaz‐Maroto et al., 2004). Drying methods can create a porous structure on the surface of plant materials and help accessibility of solutes, such as EOs, through enhancement of mass transition coefficient during extraction, therefore higher volatile compositions can be obtained than those from fresh samples (Feyzi et al., 2017). Our results were not in line with the results of previous research done by Samadi et al. (2018) and Ghasemi Pirbalouti et al. (2013) where dried plants of *Dracocephalum kotschyi* and basil landrace, respectively, had lower content of monoterpene hydrocarbons than fresh samples. This may possibly be related to the type and source of the plants, as well as the conditions applied for drying techniques (Shahhoseini et al., 2013). Similar to our study, it was found that microwave‐drying significantly decreased hydrocarbon monoterpene compounds (Hazrati et al., 2018; Mohammadizad et al., 2017).

The sesquiterpenes content was also affected by the drying technique employed. The results for hydrocarbon sesquiterpenes indicated that microwave and oven‐drying (60 and 80°C) methods significantly decreased these compositions. The maximum content of δ‐cadinene was produced by the freeze‐, oven‐ (40°C), and shade‐drying methods; nevertheless, its amount was significantly reduced by increasing temperatures using microwave and oven (60 and 80°C). The maximum amounts of α‐copaene (13.7%), germacrene‐D (9.5%) and E‐caryophyllene (5.2%) were obtained when freeze‐drying was used, displaying a notable enhancement compared with other drying methods (Table 1). Generally, sesquiterpenes have higher molecular weight than monoterpenes, and therefore, they are less fugacious and less easily removed from the plant material; as respects, they are sensitive to oxidation response and subjecting the samples to extended drying times would decrease the sesquiterpene compositions (Chua et al., 2019).

Spathulenol, α‐cadinol, intermedeol, guaia‐3,10(14)‐dien‐11‐ol, and epi‐α‐bisabolol were recognized as the dominant constituents of oxygenated sesquiterpenes, among which the highest content (54.1%) was related to spathulenol in plants dried under sunlight. Because to their higher polarity, oxygenated sesquiterpenes have higher boiling points compared to hydrocarbon sesquiterpenes. Hence, these compositions do not evaporate easily under sun‐ or oven‐drying. Previous researchers have reported similar results to those obtained in the current study; the increase of oxygenated sesquiterpenes in aromatic plants treated at high temperatures (Hazrati et al., 2018; Rezazadeh et al., 2006; Saeidi et al., 2016).

**FIGURE 3:**
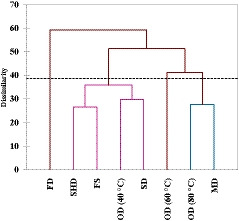
Dendogram obtained by hierarchical cluster analysis of essential oil value and compound from dried samples of *Stachys lavandulifolia*. FD, freeze‐dried; FS, fresh sample; MD, microwave‐dried; OD, oven‐dried; SD, sun‐dried; SHD, shade‐dried

A dendrogram of cluster analyses, using EO value and compound, separated the different drying methods into four groups (Figure 3). The microwave‐ and oven‐drying (at 60 and 80°C) methods were placed in the first group, while the sun‐ and oven‐ (at 40°C) drying methods were placed in the second group. The fresh and shade‐drying methods were placed in the third group. Freeze‐drying methods was placed in the separate group. α‐Terpinene and p‐cymene led to classification of the oven‐ (60 and 80°C) and microwave‐drying methods in the first group, while δ‐cadinene, spathulenol, and α‐cadinol were the most important compounds responsible for existence placed in the second group.

### 3.3 Effect of drying techniques on antioxidant activity

The antioxidant capacity of *S. lavandulifolia* EO was evaluated by two supplementary in vitro measurements, which demonstrated the IC_50_ contents of the assayed EOs determined in DPPH and RP assays. The outcomes indicated that all EOs studied had significant antioxidant attributes, and additionally that drying techniques effect affirmatively the antioxidant power of *S. lavandulifolia* EO (Table 2). In fact, the best portion in the DPPH and RP measure was attained by the EOs acquired from *S. lavandulifolia* when freeze‐dried (IC_50_ amounts 101.8 ± 0.8 mg/ml and 315.2 ± 2.1 mg/ml, respectively), followed by those extracted from the samples dried in shade (IC_50_ amounts 103.3 ± 0.5 mg/ml and 321.2 ± 2.3 mg/ml, respectively). The minimum activities were registered by the oven‐dried samples (at 80°C) with IC_50_ amounts of 180.3 ± 2.7 mg/ml and 495.2 ± 3.8 mg/ml, respectively. The EOs acquired from samples dried in the microwave indicated intermediate antioxidant activity (IC_50_: 120.0 ± 1.1 mg/ml and 340.5 ± 3.2 mg/ml, respectively). In addition, as anticipated, none of the EOs studied were as strong as butylated hydroxytoluene (BHT) (IC_50_: 24.5 ± 0.1 mg/ml and 45.6 ± 0.2 mg/ml, respectively).

**TABLE 2:** IC50 values (mg/ml) of *Stachys lavandulifolia* essential oils in different drying methods and BHT

Antioxidant tests	Essential oils								Standard antioxidant
	FS	SHD	SD	FD	MD	OD (40°C)	OD (60°C)	OD (80°C)	BHT
DPPH assay	139.2 ± 1.9	103.3 ± 0.5	122.2 ± 1.6	101.8 ± 0.8	120.0 ± 1.1	108.2 ± 1.5	145.0 ± 2.3	180.3 ± 2.7	24.5 ± 0.1
Reducing power assay	435.5 ± 2.9	321.2 ± 2.3	350.6 ± 2.8	315.2 ± 2.1	340.5 ± 3.2	330.0 ± 2.5	460.2 ± 3.3	495.2 ± 3.8	45.6 ± 0.2

Note

Values are given as mean ± *SE* (*n* = 3).

Abbreviations: BHT, butylated hydroxytoluene; FD, freeze‐dried; FS, fresh sample; MD, microwave‐dried; OD, oven‐dried; SD, sun‐dried; SHD, shade‐dried.

The current data show that the use of drying techniques has an affirmative effect on the antioxidant power of *S. lavandulifolia* EO. There is not a great deal of extant information on the influence of various drying methods on the antioxidant activity of *S. lavandulifolia* EO. Commonly, the antioxidant powers of plant EOs were ascribable to their chemical combination and particularly to their main compositions. Therefore, the various levels of the antioxidant attributes found for the investigated EOs extracted from *S. lavandulifolia* samples dried with various techniques may be ascribed to the low change of their various main compositions, especially the amounts of spathulenol and myrcene. Apparently, there exists a consensus that the antioxidant attributes of EOs cannot simply be described by the practice of their main compositions (Ahmed et al., 2018). Other studies have shown that spathulenol, E‐caryophyllene, and germacrene‐D could also be responsible for the antioxidative activity reported (Baschieri et al., 2017; Cutillas et al., 2018; Sarikurkcu & Ćavar Zeljković, 2020).

## 4 CONCLUSION

Our findings showed that transformations occurred in EO compounds of *S. lavandulifolia* during drying related to the drying technique and temperature employed. The amounts of bioactive substances in fragrant plants are related to drying technique and temperature, in addition to the biological and morphological characteristics of the plants. As well as the above, the results showed that drying under oven (60 and 80°C) caused a decrease in EO value, while drying by shade resulted in the greatest amount of EO. Both microwave‐ and oven‐drying considerably decreased α‐pinene, β‐pinene, and myrcene amounts. Thus, depending on the flavored composition, one of the drying techniques can be used. Overall, shade‐ and sun‐drying were capable of producing the optimum values of EO and spathulenol, respectively.

## CONFLICT OF INTEREST

The authors declare that they do not have any conflict of interest.

## ETHICAL APPROVAL

This study does not involve any human or animal testing.

## INFORMED CONSENT

Written informed consent was obtained from all study participants.

## Data Availability

Research data are not shared.
